# Thoracoscopic removal of oesophageal duplication cyst

**DOI:** 10.4103/0972-9941.78350

**Published:** 2011

**Authors:** Prakash Agarwal, Rajkishore Bagdi

**Affiliations:** Department of Paediatric Surgery, Apollo Children’s Hospital, Shafee Mohammed Road, Chennai, India

**Keywords:** Oesophageal duplication cyst, thoracoscopy

## Abstract

A 4-year-old boy presented with vomiting and recurrent cough. He was investigated and found to have thoracic oesophageal duplication cyst. He was taken up for thoracoscopic removal of the cyst. The cyst was attached to the oesophagus and shared a common wall. The boy tolerated the procedure well and follow-up showed no recurrence of the cyst with total resolution of the symptoms. We share our experience with the management of this boy.

## INTRODUCTION

Developmental disorders of ventral budding of the lung primordium from the embryonic foregut may present as cystic duplications or diverticuli of the bronchus or oesophagus.

Resection of mediastinal cysts is often indicated for definitive diagnosis, to alleviate symptoms and to prevent the development of associated complications.

Open resection by thoracotomy has been the only definitive treatment for these lesions in the past, but in view of their benign histology, a less-invasive approach is desirable. Growing experience and improved instrumentation for small patients have broadened the repertoire of procedures that can be accomplished by paediatric minimal-access surgery (MAS).

There have been previous reports on video-assisted thoracoscopic resections of benign mediastinal cysts. However, there has only been few reports on the resection of an oesophageal cyst using this approach.[1,2] We have performed thoracoscopic resection of an oesophageal cyst with minimal chest wall trauma and a favourable outcome in a young patient.

## CASE REPORT

**Figure 1:**
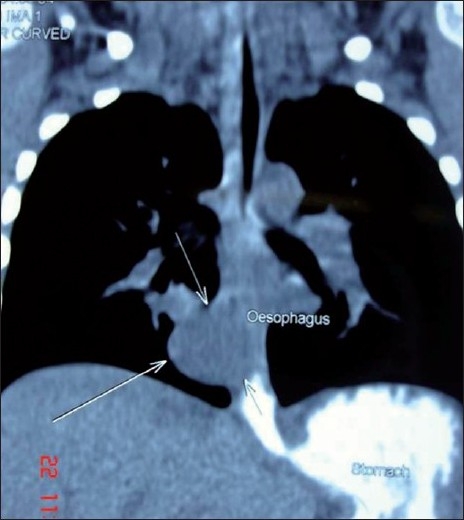
Axial reconstruction of the CT scan showing well-circumscribed cystic mass closely related to the oesophagus.

A 4-year-old boy was previously well presented to us with 3-month history of vomiting and intermittent cough. Chest X-ray films showed a mild haziness in the right cardiac border. A CT scan of the thorax revealed a well-circumscribed cystic mass closely attached to the oesophagus, just above the diaphragm [Figure 1]. The mass was not enhanced by the use of intravenous contrast medium. Oral contrast medium did not show communication of the mass with the oesophagus. Upper GI endoscopy showed mild indentation of the oesophagus by the cyst. The patient was otherwise clinically well. Preoperative clinical diagnosis was a benign enteric or oesophageal cyst. Surgical resection of the cyst was recommended and video-assisted thoracoscopic surgery was offered as an alternative to the conventional thoracotomy approach.

## MATERIALS AND METHODS

**Figure 2:**
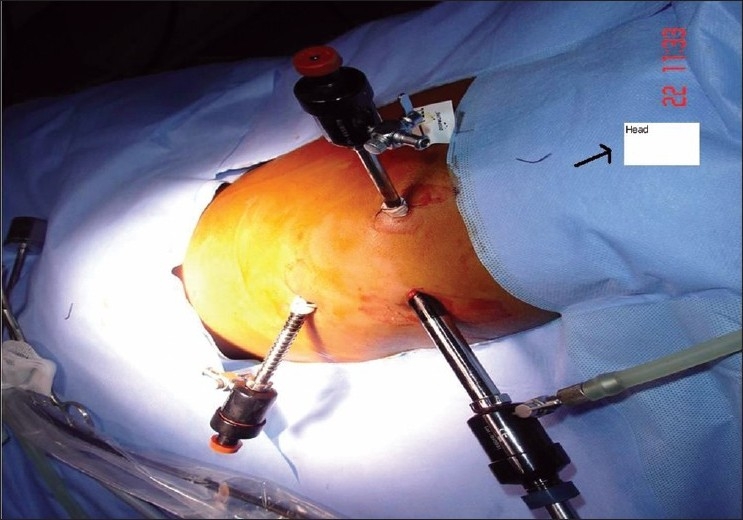
Lateral decubitus position with port placement and the head end marked.

**Figure 3:**
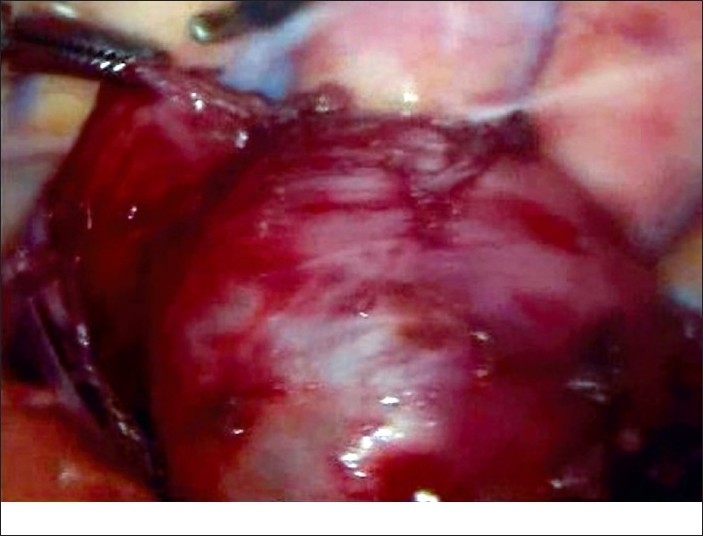
Dissection of cyst showing no separate plane between cyst and oesophageal wall.

With the use of general anaesthesia and endotracheal intubation, the patient was placed in the full left lateral decubitus position. A nasogastric tube was put into place. The patient was prepared and draped as for a thoracotomy. A 10-mm 30º telescope was inserted through the fifth intercostal space at the posterior axillary line. Two instrument ports were created, at the fourth intercostal space, below the tip of scapula and at the seventh interspace just in front of the posterior axillary fold [Figure 2]. The mediastinal pleura over the base of the cyst was circumferentially incised. The cyst had a wide base and no division plane separating it from the underlying oesophagus was found [Figure 3]. We deliberately avoided dissection of the cyst off the oesophagus in fear of perforation. A decision, therefore, was made to open the cyst and examine it from inside. We passed a flexible bronchoscope into the oesophagus for illumination to rule out any perforation and facilitate dissection. The cyst was incised which contained approximately 20 ml of mucosy fluid. The cyst wall was about 0.5 cm thick with smooth mucosal lining inside. No communication with the oesophagus was found. The wall was excised except for a small island (l×lcm) of the cyst wall was left on the oesophagus, the mucosa of which was cauterized. A 20 Fr chest-drain tube was left in place.

## DISCUSSION

Ventral budding of the lung primordia from the foregut occurs at 3-4 weeks of gestation. Aberrations in this process during this or subsequent stages of development may result in duplications of the oesophagus or bronchi. Simple oesophageal or bronchial duplication cysts are most commonly found in the mediastinum.[3,4] Histologically, they may be differentiated by the presence of cartilage in the wall of a bronchial cyst and the presence of two well-defined muscle layers in an oesophageal duplication.[5] The common embryological origin of these structures is substantiated by the fact that the lining epithelium of both lesions may be ciliated respiratory epithelium.

The diagnosis of bronchogenic cyst cannot be made solely based on a lung radiograph. In 69% of cases, it requires a CT scan and in 100%, it depends on an MRI that enables distinction between cystic and tissue mass.[6] Endoscopic scanning can be useful to document a paraoesophageal cyst.[7] Precise localization of the cyst may prompt a modification of the patient’s position on the operating table. Lateral decubitus is the usual position for such procedures, but the patient could be tilted forward or backward if necessary.

These cysts are histologically benign and some controversy exists regarding the need to excise asymptomatic cysts in adult patients. Complete excision is the preferred treatment in children because of the high risk of obstructive respiratory problems, and also because these cysts do not regress spontaneously and occupy space destined for growing respiratory tissue. Malignant transformation, although rare, has been described in children and adults.[8] Our patient had obstructive symptoms of vomiting and cough for which he was worked up and found to have the cyst on CT scan.

Treatment of thoracic duplication cyst consists primarily of excision, and this may be accomplished thoracoscopically, although this may not be always possible. For thoracoscopic approaches, at least three and occasionally four ports are required.[9] Reports of thoracoscopic removal of mediastinal cysts in adults[10,11] and children[12] have been limited to one or two cases. Merry *et al*, in their report of eight cases in children ranging from 3.5 months to 8 years had an average operating time of 106 min with six cases having complete cure and all cases discharged by 3 days with minimal pain. One patient had a recurrence due to incomplete resection which was managed by open thoracotomy and one patient developed pneumothorax as chest drain was not inserted postoperatively. The patient recovered with the insertion of chest drain. They conclude from this experience that many simple foregut duplications may be excised safely by MAS in children. Two technical points are noted: (a) central mediastinal lesions require chest-tube drainage postoperatively; and (b) complete excision is required to avoid recurrence.[2] Hirose *et al*, in a series of six cases of foregut duplication cyst managed thoracoscopically report short operating time, improved cosmesis and less pain obviating the need for narcotics. In their series most patients had a short hospital stay with tube thoracostomy required for less than 12 hours. They suggest thoracoscopic resection should be considered the standard of care for resection of these benign cysts.[13]

Technically they are best approached through the side of the thorax into which they protrude. Oesophageal duplications are often surrounded by oesophageal muscle which should be incised longitudinally to expose the cyst.[14] Decompression of the cyst may assist in removal, but often the dissection is aided by leaving the cyst intact as long as possible if the cyst is small.

Cyst in the wall of the oesophagus can be removed by opening the oesophageal muscle and performing an extramucosal excision of the cyst, leaving the oesophageal mucosa intact. After thoracoscopic removal the surgeon must be sure that the oesophageal lumen was not entered by inserting fluid or air in the lumen. An unrecognized oesophageal leak will lead to mediastinitis and severe morbidity.[9] Aspiration or sclerosis of these lesions is not recommended because the mucosal surface will regenerate and produce a recurrent mass. The risk of development of malignancy in the mucosa must always be considered.[14]

For thoracoscopic procedures, single lung ventilation will often facilitate the resection. Double lumen endotracheal tubes are available down to 26 French calibre, which may be used on children of over 25 kg. In smaller children or infants, mainstem intubation of the contralateral bronchus or placement of a balloon catheter (bronchial blocker) will allow deflation of lung to facilitate exposure. Selective ventilation is most useful when the lesion is located entirely in the parenchyma and less useful when it is mediastinum.[14]

In cases where the resection is incomplete electrocoagulation or laser destruction of the residual wall can be used to destroy the patent pathological cells, thereby significantly reducing the recurrence risk.[2,6,9] Careful midterm and long-term postoperative observation needs to be carried out at shorter intervals, because recurrences have been described as long as 10–25 years after the initial resection. All of these recurrences were managed by thoracotomy.[15]

Thoracoscopic resection of an oesophageal cyst in a young child is technically feasible, associated with minimal chest wall trauma and has a favourable outcome.
